# Feeding Problems Among Typically Developing Children From India Aged 1 to 5: A Cross-sectional Study

**DOI:** 10.1097/DBP.0000000000001402

**Published:** 2025-07-28

**Authors:** Vyshnavi Nelliyottu Kuniyil, Srikanth Nayak, Rakesh Chowkalli VeeraBhadrappa, Suneel C. Mundkur, Usha Devadas

**Affiliations:** * Department of Speech and Hearing, Manipal College of Health Professions, Manipal Academy of Higher Education, Manipal, Karnataka, India;; † Department of Audiology and Speech-Language Pathology, Yenepoya Medical College, Yenepoya University (Deemed to be University), Mangalore, Karnataka, India; and; ‡ Department of Paediatrics, Kasturba Medical College, Manipal Academy of Higher Education, Manipal, Karnataka, India.

**Keywords:** neurotypical children, feeding difficulties, prevalence, mealtime behavior, child nutrition

## Abstract

**Objective:**

Feeding problems in typically developing children (TDCs) are quite common in early childhood and are of great concern for parents, as they significantly impact nutritional intake and overall health. This study aimed to evaluate the prevalence of feeding difficulties in typically developing children from India aged 1 to 5 years using the Kannada version of the Behavioral Pediatrics Feeding Assessment Scale (BPFAS).

**Method:**

Data were gathered from 253 parents of typically developing children via a purposive sampling method. The parents completed a self-reported demographic questionnaire and a Kannada version of the BPFAS.

**Results:**

The survey analyzed the responses of 124 (49%) male and 129 (51%) female children. According to the BPFAS cutoff scores, the prevalence of feeding difficulties among TDCs in the study was 28.9% (n = 73) (TFS >84) and 18.2% (n = 46) (TPS >9). Even though a higher percentage of children exhibited negative feeding behaviors, very few parents considered these behaviors problematic for them. Furthermore, analysis revealed a weak negative correlation between BMI and TFS and TPS scores (Pearson r values of −0.027 [*p* = 0.669] for the TFS score and −0.049 [*p* = 0.436] for the TPS score).

**Conclusion:**

The present study reveals a higher prevalence of parent-perceived feeding issues in young, typically developing children from India. This highlights the necessity of educating Indian parents about feeding issues in young children, their potential long-term consequences, and the need for early intervention.

## INTRODUCTION

Feeding is a complex and highly coordinated multisystem function that enables the safe and efficient movement of food or liquid from the mouth to the stomach.^1^ Most typically developing children (TDCs) acquire feeding skills without difficulty. However, feeding issues/problems are not uncommon among typically developing toddlers or preschoolers.^2,3^ Feeding problems, especially during early childhood, can significantly affect a child's growth, nutritional intake, and overall development.^4^ These difficulties typically emerge between 6 months and 4 years, a critical period for developmental transitions from liquid to solid foods.^5^

Feeding problems in young children arise from multiple factors (medical, nutritional, developmental, and psychological), making clear definitions challenging due to their overlap. Factors such as parent-child interaction patterns^6,^ disordered maternal feeding behaviors,^7^ and the overall feeding environment^8^ can all exacerbate feeding issues in children. Cultural and environmental factors add further complexity, as feeding behaviors considered pathological in 1 region may be viewed as normal elsewhere. Hence, a variety of terminologies are used in the literature to describe feeding difficulties, such as “feeding problems,” “food refusal,” “selective eating,” “food selectivity,” “picky eating,” “fussy eating,” and “dietary restrictions.” These inconsistent terminologies challenge the ability to distinguish normal developmental behaviors from pathological conditions, resulting in wide variability in reported prevalence estimates.

Various studies have reported that the prevalence of feeding difficulties in TDCs ranges between 4% and 45%, with most studies indicating a range of 20% to 30%.^9–13^ Severe feeding issues are less common, affecting approximately 1% to 2% of TDCs.^5,9–16^ A recent review revealed that the prevalence of Avoidant Restrictive Food Intake Disorder (ARFID) in nonclinical samples ranges from 0.3% to 15.5%, with higher rates observed in clinical populations.^17^ Emerging data on Pediatric Feeding Disorder (PFD) suggest a prevalence ranging from 20% to 30%, reflecting its broader diagnostic criteria.^18^ However, these prevalence estimates must be interpreted cautiously due to variability in characteristics and assessment methods.^5,10,15^ In addition, regional differences in diet, culture, and feeding practices further influence these variations, as definitions of “feeding problems” may differ according to local norms and expectations.^5,11,12,14,15^

Children with feeding difficulties are generally classified into 3 fundamental categories: those with restricted appetite, those with selective intake, and those with food neophobia.^11^ While most TDCs acquire feeding skills without significant issues, transient eating problems may arise during early childhood. If these issues persist, they can develop more serious feeding problems.^19^ The transition to solid foods is particularly challenging and is often marked by food refusal or selective eating patterns. Although common, such behaviors can lead to nutritional deficiencies, impaired growth, and adverse developmental outcomes.^20^ Since parents serve as the primary gatekeepers of a child's diet, their perceptions of and responses to feeding issues play a critical role in managing these difficulties.^21^

Assessing feeding difficulties in young children requires a thorough multidimensional approach, including medical history, physical examination, evaluation of muscle tone and coordination, and analysis of the feeding environment.^22^ Several tools have been developed to evaluate feeding across age groups and conditions. One widely used tool is the Behavioral Pediatric Feeding Assessment Scale (BPFAS),^23^ a parent-report questionnaire designed to evaluate mealtime and feeding behaviors in young children. The BPFAS is a simple tool to administer and has demonstrated high reliability and specificity. It has been validated in multiple studies and extensively used in global research.^23–26^

In India, where cultural practices and dietary habits differ widely, data on feeding difficulties in preschool-aged children remain limited. Given the broad spectrum of contributing factors and the serious potential consequences of persistent feeding problems, understanding their prevalence in different cultural contexts is crucial. Hence, the present study aimed to identify the prevalence of feeding problems in children from India (TDCs) aged 1 to 5 years.

## METHODOLOGY

### Participants and Study Settings

The present study was conducted between June 2023 and April 2024 in the Department of Pediatrics at a tertiary care hospital, as well as at Anganwadi centers and kindergarten schools in and around the Udupi district, India.

The study population comprised parents or primary caregivers of TDC aged 1 to 5 years. Potential participants were screened using the International Classification of Functioning, Disability, and Health (ICF) checklist, a tool developed within the framework of the WHO's ICF model. This model assesses an individual's functioning and disability, considering environmental and personal factors, as well as functioning and impairment in terms of participation, activities, and physical structure. Children were excluded from the study if they were born preterm (before 37 weeks' gestation) or had a documented history of cleft lip/palate, autism spectrum disorder (ASD), attention-deficit/hyperactivity disorder (ADHD), intellectual disability (ID), developmental delays, or other neurological or developmental disorders. Only children meeting all screening criteria without any developmental or neurological concerns were classified as typically developing and included in this study.

A purposive sampling approach was used to recruit participants for the study. The required sample size of 288 was determined on the basis of the results of a prior pilot study. (Prevalence (P) = 0.25 (based on 3 of 12 children with feeding problems in the pilot study), confidence level = 95%, absolute precision (d) = 0.05.)

### Data Collection

This study protocol was reviewed and approved by the Institutional Research Committee and the Institutional Ethics Committee (IEC:301/2023). For the data collection, the researcher approached parents/caregivers of potential participants at pediatric outpatient clinics during immunization visits at a tertiary care hospital, as well as at Anganwadi centers and kindergartens during child drop-off and pick-up times. The researcher explained the purpose of the study, and parents were invited to participate. Written informed consent was obtained from all parents who agreed to participate in the study.

Data collection began with gathering demographic (age and gender), developmental (motor and speech and language), and medical (any comorbid conditions) details of the children from the consenting parents. The height of the children was measured via a stadiometer, and their weight was measured via a digital weighing scale. The body mass index (BMI) of an individual is calculated as their weight in kilograms divided by the square of their height in meters via the World Health Organization (WHO) Anthro software.^27^ Finally, parents of TDCs were requested to complete the Kannada version of the BPFAS as per the instructions provided in the questionnaire.

### Behavioral Pediatrics Feeding Assessment Scale (BPFAS)

The BPFAS, a parent-reported questionnaire, consists of 35 items divided into two sections. The first section included 25 items designed to assess the child's behavior during mealtime, whereas the remaining 10 questions assess the parents' perceptions about their child's feeding patterns and mealtimes at home and the strategies adopted by them to address them. Parents are requested to answer each question indicating how often a behavior is observed on a five-point Likert scale (1 indicated never, 3 indicated sometimes, and 5 indicated always) and whether they consider these behaviors problematic for them, indicating “Yes” or “No.” The responses obtained were analyzed as Total Frequency Score (TFS) (maximum score 175) and Total Problem Score (TPS) (maximum score 35). Children with a TFS score of more than 84 and a TPS score of 9 are considered to be at risk for feeding problems. The present study translated the BPFAS into the Kannada language with prior permission from the developer author using standard forward-backward translation methods involving bilingual experts.^28^ The final version was reviewed and approved by the developer author to ensure accuracy and cultural relevance.

### Statistical Analysis

The data were analyzed via Jamovi software version 2.3.28. Frequencies and percentages were used to summarize categorical variables, whereas means and standard deviations (SDs) were used to summarize continuous variables. The Pearson χ^2^ test was employed to investigate the correlation between feeding problems and demographic and anthropometric variables. Finally, Pearson correlation coefficient was calculated to test the correlation between raw TFS and TPS (regardless of whether they exceeded the relevant cutoff) with BMI.

## RESULTS

The researcher personally reached out to 288 parents of TDC aged 1 to 5 years in outpatient pediatrics, Anganwadi centers, and preschools. Of these, 253 parents (87.84%) agreed to participate and successfully completed the Kannada BPFAS. As shown in Table 1, the responses from the parents were obtained for 124 (49%) male and 129 (51%) female children, with an average age of 3.36 ± 1.30 years. The distribution of children across age groups was as follows: 17.0%; n = 43 (1–2 years), 16.6%; n = 42 (2–3 years), 17.8%; n = 45 (3–4 years), 48.6%; and n = 123 (4–5 years). Most the respondents were mothers (n = 224; 88.5%), whereas 11.5% (n = 29) were fathers.

**Table 1.** Demographic (Child and Parent), BMI Scores, and BPFAS Scores of the Participants

	Mean ± SD
Child's age (yr)	3.36 ± 1.30
BMI score	16.18 ± 5.92
TFS score (total)	76.5 ± 15.0
TPS score (total)	4.16 ± 5.53
Parental age (yr)	33.1 ± 5.18
	N (%)
Gender	
Male	124 (49.0)
Female	129 (51.0)
TFS score by cut-off (>84)	73 (28.9)
TPS score by cut-off (>9)	46 (18.2)
Parental gender	
Male	29 (11.5)
Female	224 (88.5)

As shown in Table 1, the mean TFS score was 76.5 ± 15.0, and the mean TPS score was 4.16 ± 5.53. However, on the basis of the BPFAS cutoff criteria, 28.9% of typically developing children (n = 73) scored above the threshold for TFS (>84), and 18.2% (n = 46) exceeded the cutoff for TPS (>9), suggesting the presence of clinically significant feeding difficulties in a substantial portion of the sample. Furthermore, feeding problems were slightly higher in female children compared with male children. Age-wise analysis revealed that children in the 4–5-year-old age group exhibited a higher frequency of feeding difficulties than younger age groups, highlighting a potential increase in feeding issues with age (Table 2).

**Table 2.** BPFAS Score for Different age Groups and Gender

	TFS Score >84	TPS Score >9
	N (%)	N (%)
Gender		
Male	34 (13.4)	21 (8.3)
Female	39 (15.0)	25 (9.9)
Age ()		
1–2	17 (6.7)	8 (3.2)
2–3	13 (5.1)	8 (3.2)
3–4	11 (4.3)	4 (1.6)
4–5	32 (12.6)	26 (10.3)

In summarizing the key aspects of the children's eating habits in the study, the frequency of food consumption was defined as the proportion of children who regularly consumed specific foods, with “regular consumption” categorized as responses rated 4 (almost always) or 5 (always) on a 5-point scale as follows: fruits (n = 168; 66.4%), vegetables (n = 183; 72.4%), milk (n = 149; 58.9%), meat/fish (n = 160; 63.3%), and starch (n = 229; 90.9%). Moreover, 63.3% (n = 160) of the children enjoyed eating, and 75.1% (n = 190) came willingly to mealtime.

The study identified a range of unhealthy eating habits frequently exhibited by the children (rated by parents almost always or always), such as leaving the table (n = 189; 74.8%), delaying eating by engaging in conversation (n = 171; 67.6%), taking longer time to finish meals (n = 208; 82.2%), exhibiting poor appetite (n = 162; 64%), negotiating about food choices (n = 187; 73.9%), eating junk food and not eating during mealtime (n = 170; 67.2%), preferring to drink rather than eat (n = 149; 58.9%), and refusing to eat but requesting food immediately after mealtime (n = 127; 50.3%). Despite the high frequency of negative feeding behaviors among children, only a small proportion of parents perceived these behaviors as problematic. This discrepancy highlights a potential gap between the actual occurrence of feeding difficulties and parental recognition of them as concerns. For example, while many children frequently left the table during meals, only 26.1% (n = 66) of parents considered this as problematic. Similarly, only 25.3% (n = 64) expressed concern over prolonged mealtime, 20.9% (n = 53) for tantrums, 19.8% (n = 50) for keeping food in the mouth, and 19.0% (n = 48) for delaying eating by talking. Even fewer parents viewed behaviors such as poor appetite (15.0%; n = 38), negotiating over food (17.4%; n = 44), preferring drinks over food (11.1%; n = 28), and refusing food only to request it shortly after (9.1%; n = 23) as problematic. These findings suggest that while problematic behaviors are common, they may be underrecognized or normalized by caregivers, potentially delaying timely intervention. Furthermore, parents' feelings about their child's feeding patterns and mealtimes at home and the strategies they adapted to address them were analyzed based on the BPFAS rating. The results revealed that 54.2% (n = 137) of parents expressed a lack of confidence in their child's nutritional intake, 54.2% (n = 137) of parents were not confident in managing their child's behavior during mealtimes, approximately 32.1% (n = 81) of parents reported experiencing frustration or anxiety while feeding the child, and 44.3% (n = 112) felt angry while feeding their child. Subsequent to this, many parents affected by their child's eating habits, employed certain feeding practices (almost always or always) to get their child to eat such as coaxing their children to take a bite (n = 197; 77.8%), preparing alternative meals (n = 214; 84.7%), threatening (n = 146; 57.8%), and force-feeding (n = 121; 48.3%). Among these strategies, the parents perceived force-feeding (n = 42; 16.6%) and coaxing (n = 35; 13.8%) as more problematic.

### Relationships Between Feeding Problems and BMI

The correlation between BMI and feeding problems (TFS and TPS scores) was examined via the Pearson correlation coefficient test. The results revealed a weak negative correlation, with Pearson r values of −0.027 (*p* = 0.669) and −0.049 (*p* = 0.436) for the TFS and TPS scores, respectively.

## DISCUSSION

The present study investigated the prevalence and characteristics of feeding problems in TDCs aged 1 to 5 years using the Kannada version of the BPFAS. The results revealed that 28.9% and 18.2% of the children scored above the clinical cutoff for TFS and TPS, respectively, indicating that nearly one-third of the TDS children experienced feeding difficulties. These findings are consistent with previous studies reporting a prevalence of feeding problems in 20% to 30% of typically developing children.^9–13^ However, the TFS and TPS scores obtained in the present study were distinct from those reported in studies conducted in New Zealand^21^ and Greece.^10^ These studies reported lower TFS and higher TPS scores than those observed in the present study, suggesting that even though fewer problematic feeding behaviors were reported by the children, parents perceived them as more problematic. In contrast, the present study revealed higher TFS scores but lower TPS scores. That is, even though children from India exhibited more frequent negative feeding behaviors, such as prolonged mealtimes, food refusal, poor appetite, and a preference for drinking overeating, only a small percentage of parents identified these behaviors as problematic, highlighting a potential disconnect between observed behaviors and caregiver perceptions. This gap in perception may stem from cultural acceptance of picky eating as a phase, or a possible difference in parental expectations or cultural attitudes toward feeding challenges. These cross-cultural differences emphasize the role of sociocultural context in shaping parental perceptions of feeding behavior and the importance of considering cultural norms when assessing and interpreting feeding difficulties.

Our study, which used the BPFAS to assess feeding problems in children from India (TDCs), revealed a mean TFS of 76.5 ± 15.0 and a TPS of 4.1 ± 5.5. These results closely align with those of an Australian study (34), which reported a similar mean TFS of 68.1 ± 15.7 and a TPS of 4.1 ± 6.2 among 54 TDCs aged 2 to 6 years. However, compared with Turkish research,^14,29^ our study reported a higher mean TFS but lower or comparable TPS scores, suggesting cultural differences in parental strategies and concerns regarding child nutrition. In addition, our study outcomes (mean TFS and TPS values) were higher than those in a Canadian study,^26^ which reported a mean TFS of 63.9 ± 14.2 and a TPS of 3.0 ± 4.5 in TDCs. These comparisons underscore the importance of considering sociocultural differences when addressing feeding problems in children.

In the present study, feeding difficulties were found to be more frequent in the 4-5-year age group than in the other age groups, suggesting that feeding challenges may become more pronounced with age. This aligns with previous research indicating that feeding difficulties can persist or worsen if not addressed during early developmental transitions from liquids to solids.^5,19^ This may be attributed to food neophobia, aversion to trying new food,^30^ environmental factors (psychological state of both the child and caregiver),^31^ and parental feeding styles.^32^ This age-related increase may also reflect growing child autonomy, increased exposure to food variety, and more complex parent-child interactions during mealtimes.^33^ Furthermore, feeding difficulties were observed at a slightly higher rate in female children than in male children. In a similar study, Esparo et al.^15^ did not find differences in feeding problems between genders. However, data comparing feeding behaviors across age groups and genders among typically developing children are limited, highlighting the need for larger-scale studies to better understand potential sex differences in feeding patterns. Although the findings of the present study reported that more than 60% of children consumed fruits (66.4%), vegetables (72.4%), and milk (59%), this percentage was below the level recommended by the WHO. This can be attributed to family eating patterns, as some families may not consume fresh fruits due to economic constraints, especially in rural areas, food preferences of children (taste and texture sensitivity), limited early exposure to these diverse foods, or reluctance to use novel foods that are less tasty. In addition, many Indian families often consume home-cooked starchy food items such as rice and wheat. This is also reflected in the present study, where 90.9% of the children often consumed starchy foods. Furthermore, approximately 82.2% of the children had prolonged mealtimes, whereas some had poor appetites, which may be due to distractions (smartphone/television usage), picky eating, and inconsistent eating routines. Other refusal behaviors observed during mealtimes include negotiation and crying, which may be due to children's reactions to parental pressure or seeking attention. These findings complement the literature, such as that of Sdravou et al.,^10^ who emphasized a decreased intake of fruits and vegetables among young children. Hence, these behaviors should be resolved early to maintain balanced nutrition.

In terms of parental perceptions, over half of the respondents reported a lack of confidence in their child's nutritional intake and their ability to manage mealtime behaviors, yet only a small percentage considered these concerns problematic. This discrepancy suggests that while feeding stress is common, it is not always acknowledged as a clinical concern. In addition, a significant number of parents reported frequent feelings of frustration, anxiety, and anger during mealtimes, which is consistent with the literature showing that feeding problems can lead to heightened parental stress and negative parent‒child dynamics^6,7.^ Furthermore, most parents reported engaging in coercive feeding practices (e.g., threats, force-feeding), as measured by BPFAS. This highlights the need to educate parents about more effective and positive feeding strategies. However, future research should clearly describe how often and in what contexts these coercive practices occur, enabling a more precise understanding of their impact.

### Feeding Problems and BMI

The additional aim of this study was to investigate how BMI correlates with feeding problems. BMI is a statistical measure that employs an individual's weight and height to approximate body fat levels and is applicable to both males and females, regardless of age. This measure serves as a means to assess whether an individual falls within the categories of underweight, normal weight, overweight, or obese.^34^

Studies in the literature have revealed a pronounced inverse association between TFS/TPS outcomes and BMI in TDCs.^10,21^ However, the present study revealed a weak, nonsignificant negative correlation between BMI and TFS/TPS scores. One potential reason for this could be attributed to the sampling method. That is, the data included children from a wide age range, from 1 to 5 years. In this age range, there may be rapid fluctuations in the growth rate and BMI, which may not be associated with feeding difficulties. This finding suggests that BMI may not always be linked to feeding problems alone. Other factors, such as genetic predisposition and physical activity, may also play a significant role in BMI. In addition, parental perceptions of feeding difficulties might not align with the actual nutritional intake or growth outcomes reflected in BMI. This finding is supported by 1 of the recent studies that reported no significant differences in BMI among children with and without feeding difficulty.^35^

In summary, the present study offered valuable insights into the feeding habits of typically developing children. However, the study has several limitations. The data were collected on the basis of parental perceptions, which would have resulted in underestimation or overestimation of their children's consumption of certain food items. Furthermore, most respondents were mothers, potentially leading to a bias in the perception of children's feeding habits, as fathers and other caregivers may have different viewpoints. In addition, information on parental working status was not collected, which may impact the quality and variety of food consumed by the children. Factors such as parents' education level, their knowledge about child feeding practices (especially among new parents), their age, income levels, and whether the child was first born or second born were not evaluated, which could have provided valuable insights. Furthermore, the study did not assess the medical conditions of the child in detail. Medical conditions such as food allergies, lactose intolerance, and gastrointestinal disorders can influence the consumption of fruits, vegetables, and milk.

Future studies on feeding problems in TDCs can use a larger population and wider age range with an adequate number of samples in each age group to determine whether the number of feeding problems increases with increasing age. The BPFAS can be used as a premeasurement and postmeasurement tool to detect changes in feeding habits in children after an intervention. The inclusion of factors such as the age of the parent, level of education, and different caregivers' perceptions (such as the father, grandparents, or daycare providers) could provide more insight into how these factors may influence feeding behaviors in children. Furthermore, assessing whether a child was first born or later born with medical conditions could provide insights into the effects of birth order and medical history on feeding difficulties and their perceptions. Hence, future studies should aim to incorporate more comprehensive data collection methods, including clinical assessments, detailed health histories, and consideration of socioeconomic factors, to provide a more complete picture of children's dietary patterns and their implications for health.
